# Developing Game-Based Design for eHealth in Practice: 4-Phase Game Design Process

**DOI:** 10.2196/13723

**Published:** 2024-11-08

**Authors:** Frederiek de Vette, Aurora Ruiz-Rodriguez, Monique Tabak, Wendy Oude Nijeweme-d'Hollosy, Hermie Hermens, Miriam Vollenbroek-Hutten

**Affiliations:** 1 Biomedical Signals and Systems Group, Faculty of Electrical Engineering, Mathematics and Computer Science University of Twente Enschede Netherlands; 2 Biomechanical Engineering Group, Faculty of Engineering Technology University of Twente Enschede Netherlands; 3 Roessingh Research and Development Enschede Netherlands; 4 Medisch Spectrum Twente Enschede Netherlands

**Keywords:** game based, gamification, game, eHealth, telemedicine, development, design, engagement, game preferences, older adults, self-management, prototyping, evaluations, creative

## Abstract

**Background:**

Games are increasingly used in eHealth as a strategy for user engagement. There is an enormous diversity of end users and objectives targeted by eHealth. Hence, identifying game content that drives and sustains engagement is challenging. More openness in the game design process and motivational strategies could aid researchers and designers of future game-based apps.

**Objective:**

This study aims to provide insights into our approach to develop game-based eHealth in practice with a case study (Personalised ICT Supported Services for Independent Living and Active Ageing [PERSSILAA]). PERSSILAA is a self-management platform that aims to counter frailty by offering training modules to older adults in the domains of healthy nutrition and physical and cognitive training to maintain a healthy lifestyle. We elaborate on the entire game design process and show the motivational strategies applied.

**Methods:**

We introduce four game design phases in the process toward game-based eHealth: (1) end-user research, (2) conceptualization, (3) creative design, and (4) refinement (ie, prototyping and evaluations).

**Results:**

First, 168 participants participated in end-user research, resulting in an overview of their preferences for game content and a set of game design recommendations. We found that conventional games popular among older adults do not necessarily translate well into engaging concepts for eHealth. Recommendations include focusing game concepts on thinking, problem-solving, variation, discovery, and achievement and using high-quality aesthetics. Second, stakeholder sessions with development partners resulted in strategies for long-term engagement using indicators of user performance on the platform’s training modules. These performance indicators, for example, completed training sessions or exercises, form the basis for game progression. Third, results from prior phases were used in creative design to create the game “Stranded!” The user plays a person who is shipwrecked who must gather parts for a life raft by completing in-game objectives. Finally, iterative prototyping resulted in the final prototype of the game-based app. A total of 35 older adults participated using simulated training modules. End users scored appreciation (74/100), ease of use (73/100), expected effectivity and motivation (62/100), fun and pleasantness of using the app (75/100), and intended future use (66/100), which implies that the app is ready for use by a larger population.

**Conclusions:**

The study resulted in a game-based app for which the entire game design process within eHealth was transparently documented and where engagement strategies were based on extensive user research. Our user evaluations indicate that the strategies for long-term engagement led to game content that was perceived as engaging by older adults. As a next step, research is needed on the user experience and actual engagement with the game to support the self-management of older adults, followed by clinical studies on its added value.

## Introduction

Digital health care apps can contribute to improved patient self-management and increased health literacy, alleviating the burden on health care professionals at the same time [1-5]. This eHealth, defined as the field at the intersection of medical informatics, public health, and business, refers to health services and information delivered or enhanced through the internet and related technologies [6]. However, maintaining adherence rates among users, related to better health outcomes [7], remains an issue [8,9]. Games, or elements of games (eg, gamification, serious gaming, game-based design, and applied games), are often used as a strategy for creating motivational concepts to stimulate engagement [10-12], thereby retaining the adherence of the end user to the objectives of these eHealth apps [13,14]. Over the past few years, these gaming strategies have evolved from a novel and experimental industry practice to a more mature field of research with applications in diverse domains [15]. As such, there is an expanding body of literature on studies toward the potential and effects of such “gamified” apps [16,17], while the broader adoption of games for eHealth is still in its infancy [18-21].

A general success formula does not exist for game-based design in eHealth, as the apps in eHealth target a diversity of specific goals and users. To develop successful gaming motivation strategies for these apps, we must overcome several challenges. First, strategies that contribute to the success of a game-based app remain hidden when the rationale behind design choices does not receive attention in the literature. We may be able to discover suitable game design strategies from these works by, for example, reverse engineering or mass analyzing contents of existing apps. However, when the actual game design itself happens within a black box [22,23], successful concepts or theories lose their motivational capacities once borrowed and applied outside of their original context. There is a need to open this black box and bring research findings into practice in a useful way, which can aid developers of game-based apps in the selection of suitable content, principles, or mechanisms.

A second challenge lies in creating game content that creates durable engagement to sustain motivation, as is crucial in gamified apps [24], to adhere to the health objectives. Often, developers seem to accomplish short-term user engagement through extrinsic reward systems [11], as is suggested by gamification practices from industry (eg, [25]), which have dominated the field [26]. Game design should, however, be created for an optimal user experience in terms of aesthetics, usability, and fun [12] and primarily be entertaining [27]. By gaining insight into the preferences of the user, content can be tailored [28] to satisfy their motivational needs [29], which contributes to an engaging experience. This insight is gained through assessing the unique properties of the targeted end user and adequately addressing them by selecting game elements that are in line with these preferences as well as carefully considering the context of use and health objectives.

The purpose of this study is to provide insight into the start-to-end design trajectory of game-based eHealth. We aim to overcome the abovementioned challenges by documenting the game design process and exploring design strategies for sustained engagement. We demonstrate this approach in a case study on an eHealth platform for older adults called Personalised ICT Supported Services for Independent Living and Active Ageing (PERSSILAA). PERSSILAA was developed to identify and counter frailty among older adults. Frailty is a condition that affects many older adults. Older adults with frailty are at increased risk for the development of disability, dementia, and hospitalization [30]. The condition is multifaceted, and the major dimensions of the decline, which often occurs gradually and goes unnoticed for a long time [31], are physical and cognitive decline as well as malnutrition [32]. This vulnerability to decline is caused by a lifestyle that lacks stimulation in the following 3 dimensions: a lack of sufficient mental stimulation, physical activity, or healthy nutrition. Fortunately, when the decline is identified at an early stage—so-called prefrailty—it can be slowed down or even reversed by offering suitable training on these 3 aspects [33].

Methods to detect prefrailty among older adults and offer them the right training have been successful but resource intensive, and the demand for this specialized care is increasing with an aging population in Europe. The PERSSILAA platform aims to enable older adults to independently work on their health targets [34]. By integrating eHealth with community-based services, care can be shifted away from institutions, allowing older adults to gain greater autonomy [35].

A particular challenge here was to sustain the motivation of the older adult for long-term use. The older adult is a generally underexplored target group in gaming [36]. We present the development of this game-based app, from initial end-user research to the final version that is ready for use in real practice.

## Methods

### Game Design Process

**Figure 1:**
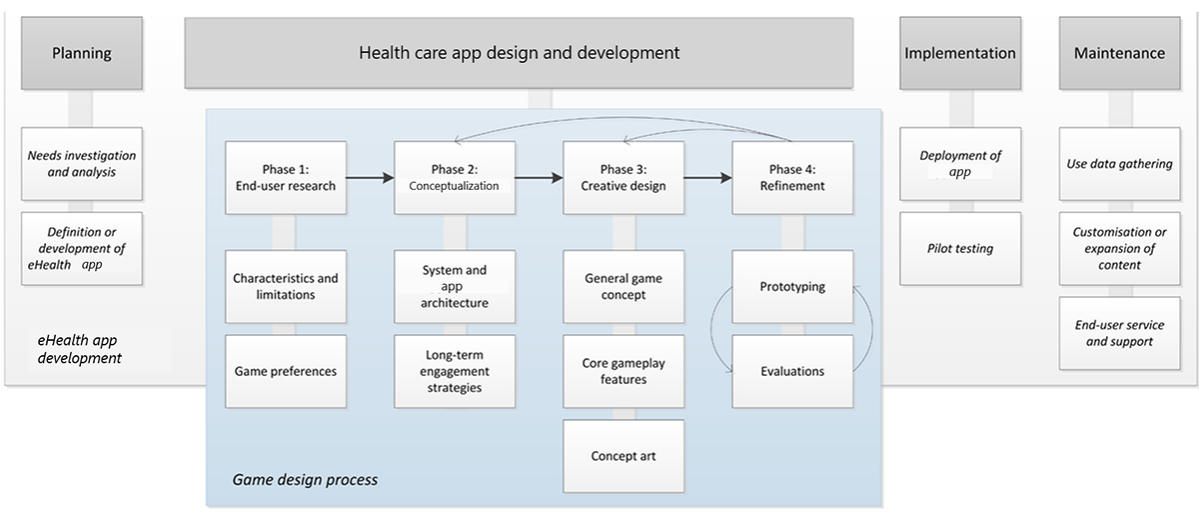
Game design process within eHealth application development.

The game design process consisted of 4 phases as part of the health care app design and development process of the eHealth app (Figure 1). These four phases were as follows:

1. End-user research phase—investigation of game preferences of the end user and specifications from the use context of the envisioned app
2. Conceptualization phase—addressing system and app architecture and the conceptual development of long-term engaging game content suitable for the end user
3. Game design phase—performing the creative and constructive processes
4. Refinement phase—prototyping and user evaluations

Each of these phases is discussed as if it had occurred chronologically, but insights gathered during subsequent phases often led to adaptations in earlier ones. Each phase consisted of several substeps, as shown in Figure 1.

For completeness, the full development cycle of the game-based eHealth app contained 3 more processes: planning, implementation, and maintenance (Figure 1). Planning took place before the health care app’s design and development. Here, the need that the envisioned eHealth app would fulfill was recognized and analyzed. Then, in implementation, the rollout of the developed gamified app for use in practice occurred. If necessary, training of primary and secondary end users (eg, caregivers and family members) also took place during implementation. Maintenance started once the app was launched and stretched the total life span of the app. It covered service and support for end users as well as back-end maintenance and keeping content up-to-date.

### Game Design Process Phase 1: End-User Research

#### End User Characteristics and Limitations

User characteristics within the context and goals of the eHealth app were explored. This included all aspects relevant to consider when creating the game design, such as computer literacy; experience with related technologies, including games; and specific usability requirements.

In the case study, we explored the characteristics of the target group eligible for the use of the gamified PERSSILAA self-management platform.

#### Recruitment of Participants

A total of 136 participants (aged between 65 and 75 years) were recruited; the inclusion criteria were sufficient computer literacy to independently use mobile devices or a PC and an interest in digital games. The participants were asked to answer a demographic questionnaire that included the abovementioned characteristics. In addition, an informed consent form for participation was provided and signed by the participants.

#### Game Preferences

The preferences of the target user toward specific game content must be investigated to be able to design engaging gamification. In previous studies, a framework to assess and classify these preferences was developed [37-40]. Assessment results in a “user profile,” subsequently translated into game design recommendations.

In the case study, we researched the specific game content that satisfies the older adult user. We reverted to a previously performed study [39], study A, and performed a follow-up [40], study B. Study A focused on investigating the general game preferences of older adults before and after providing them with a tablet with modern games to play; these preferences were assessed through questionnaires and a semistructured interview. Study B investigated the game preferences of older adults in a situation related to the use context of PERSSILAA; demographics, gaming behavior, and game preferences were measured through questionnaires. Perception and appreciation of the users regarding the game content presented in the gamified app were measured using a 1-5 visual analog scale (VAS) and semistructured interviews. The results provided an overview of game preferences of older adults regarding their gaming behavior and preferences based on their current, prior, and recent experience with video games and games. In addition, we further explored our findings by assessing game preferences before and after several weeks of using a gamified eHealth app developed specifically for this target group. The games and the gamified app were previously unknown to the participants.

### Game Design Process Phase 2: Conceptualization

#### System and App Architecture

As nongamified app development occurs in parallel, its system architecture is analyzed to determine the role and extent of the game-based design within this architecture.

In the case study, the development of the underlying, “standard” eHealth app occurred in parallel with the 4 phases of the gamification design process. The system architecture was charted in close cooperation with the back-end developers of the app. First, we decided on the extent of autonomy of the user to choose to use or disable sections of the gamified app and investigated the possibilities to implement these functionalities into both interfaces (gamified and standard). Second, we determined the possible variations or restrictions on the functionalities of the app in cases where specific (negative) health advice is given to end users. Third, milestones were identified for the inclusion of functionalities from the standard eHealth app in the gamified version at several moments in time.

#### Long-Term Engagement Strategies

To support and sustain user engagement over a predetermined amount of time, the components of the standard eHealth app that should be represented in the gamified version for meaningful interaction must be identified. This is a recommended second step in the conceptualization phase.

In the case study, we investigated how to quantify the users’ performance on the underlying health care objectives. This performance needs to be measured, as it plays a key role in strategies to sustain the engagement of users with the app throughout its entire use time. The activities that enable measuring the progress of users serve as input for the gamified app and eventually result in game content.

Developing partners in 3 domains of expertise (physical, cognitive, and nutritional health) contributed to the realization of the functionalities and services of the eHealth app. To do this, we identified the actions within each of these 3 domains that were key to the performance of users as they indicated their training progress. These actions, which we called “performance indicators,” had to be reflected through game content to support engagement. We quantified this progress with abstract “game units” (GUs). This level of abstraction allowed for the reflection of personal performance and the possibility of constructing a system that accommodates compensation between the progress of different users to create a fair and even gaming experience.

### Game Design Process Phase 3: Creative Design

In this phase, the identified elements are concretized into the design of a meaningful user experience, including aspects such as gameplay and storyline, using the knowledge gathered in phase 1. The result of this phase is the creative concept of the gamified app, often referred to as the “high concept.”

**Figure 2:**
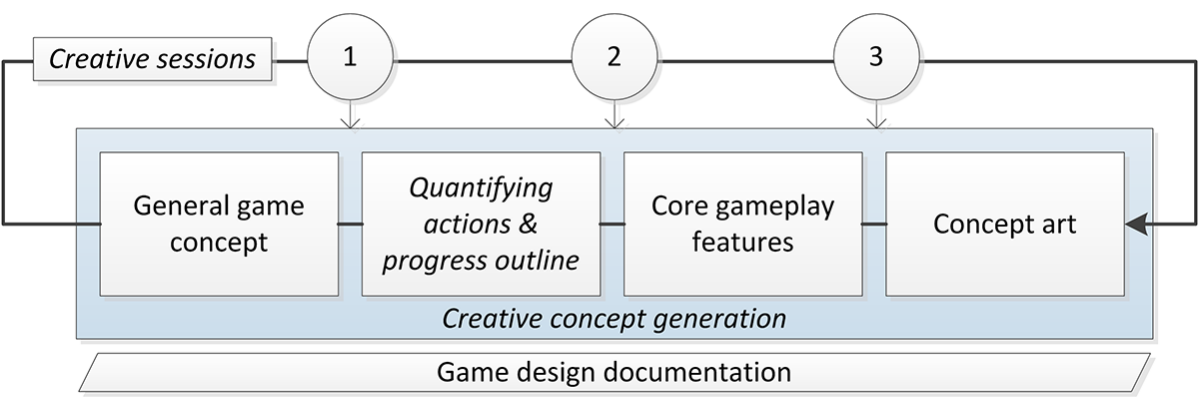
Game design phase (as applied in the case study).

We subdivided creative design into three key topics: (1) the general game concept; (2) core gameplay features; and (3) concept art, covering the visual and auditory outline of the game. We approached the game design phase as a cyclical idea generation process supported by creative sessions (Figure 2). In addition, brainstorming sessions served to reflect on and generate new insights and ideas. Quantifying performance indicators and outlining the game’s progress were addressed separately.

During this phase, the game design documentation is created [41]. This is a living document, accessible to all members of the developing team, that is continuously edited and updated. The document covers all aspects important to the development of a game, describing the vision, contents, and planning stages of prototyping and implementation as well as any outsourcing plans.

In the case study, 3 larger sessions took place during the creative design phase to support the development of the creative concept by the game designer and to ensure optimal integration of all stakeholders’ interests. In the first session, all developing partners of the PERSSILAA project participated in the creative concept for the gamified app. In the second session, we focused on ideation around feedback and reward systems; core gameplay elements such as discovery, item collection, and level progression; and ideas for the central theme. In the third session, validation regarding the suitability of specific game elements was questioned based on the insights of participants within their own expertise and experience with the target group, research, and related technologies.

### Game Design Phase 4: Refinement

#### Overview

**Figure 3:**
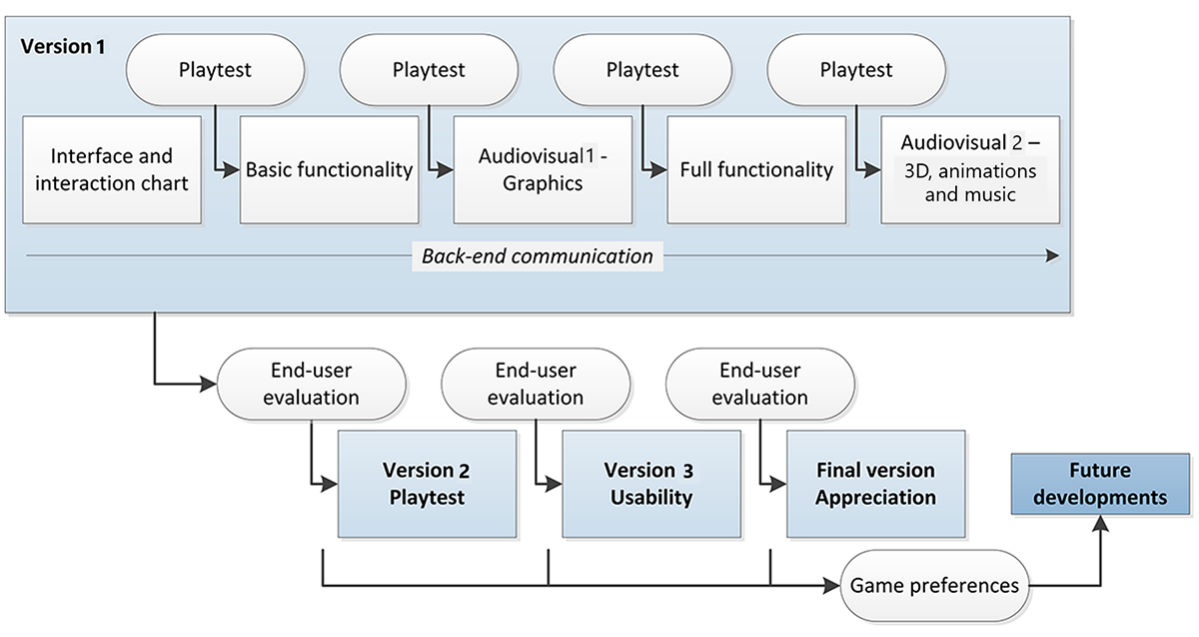
Refinement phase (as applied in the case study).

The last phase of the gamification design process covers the development of the prototype. This occurs in several stages, aided by a series of evaluation sessions. In Figure 3, we illustrate this phase, as applied in the case study, in a simplified form along with its major milestones.

#### Prototyping

As the fidelity of the prototype evolves from low to high, the back end, functionality, and visual features mature until a fully functional prototype is created. In early development, refinement iterations are more rapid and substantial.

In the case study, 4 main versions were created as predetermined by milestones set in the conceptualization phase. Here we explain version 1, which encompassed a realization of the game that was increasingly functional as well as graphically attractive. The development team and researchers thoroughly tested (“playtesting”) each iteration of the first prototype to evaluate game design and usability and eliminate bugs and design flaws at an early stage. This first version of the game was then used in an evaluation session with end users.

#### Evaluations and Data Analysis

##### Overview

Once the prototype is complete, it should undergo end-user evaluations. The goal of these sessions is to validate the current version of the system.

In the case study, 3 end-user evaluation sessions took place. All sessions delivered the following input for the succeeding version of the prototype: (1) information on end users regarding demographics, current gaming behavior, and past experience with games and devices; (2) perception and appreciation of the game content that was present in the game; and (3) information on technology acceptance, perceived motivation, and usability. The data on game preferences were gathered to follow up on our research as presented in phase 1 and to refine and expand the user profile of the older adults. The domains *discord*, *dedication*, *novelty*, *social*, *intensity*, and *threat* apply to this game concept and are used to describe the preferences of the users for specific game content on a linear scale.

##### Session 1

The first sessions focused on playtesting the gamified app with the end users. The aim was to gather feedback on the game concept, playability, and appreciation of the diverse game mechanics and the expected added value of the use of the game over the use of the standard app.

Participants were recruited by Roessingh Research and Development (Enschede) for inclusion in PERSSILAA and informed in an information meeting. Participants were instructed on the use of both versions of the app. An exclusion criterion was applied in case of health issues, limiting the use of the original app’s training modules. This criterion was automatically fulfilled, as participants in the standard app were previously assessed and classified as prefrail.

Participants used the app, basic or gamified, in their home environments. Participants were asked to log their experience with the game and to keep track of any issues that might occur. For troubleshooting and tracking game use, log data were gathered. After a period of 6 weeks, participants were asked to fill out a web-based questionnaire on their experiences. An option was also included to elaborate on the choice to not use the game.

A questionnaire, including demographics and information on gaming behavior and experience with devices and games, was gathered. The questionnaire included questions on the theme of the game; the gameplay; the graphical style; and the used game mechanics, such as the game progress. Furthermore, questions were asked about the clarity and use experience of the gamified interface compared with the basic app. These aspects were measured on 34 items rated on a 5-point Likert scale. Game preference (perception and appreciation) was measured by means of 2 times 17 statements on the content present in the game.

##### Session 2

In the second session, we evaluated the game concept, expected use, and motivation for the game in the long term. We measured the perception and appreciation of the presented game content by the user. This session focused on improving the usability of the game with video recordings and the “thinking aloud” method.

Participants (aged between 65 and 75 years) were recruited; this was done with an information letter spread by Twentse Zorgacademie (the testing and training center for care technology), and the test location was set at Twentse Zorgacademie Living Lab, Enschede. Participants were excluded from the study when they had no experience with using a PC or when they were not interested in the use of the technology used in PERSSILAA. As the physical exercises from the PERSSILAA app were not part of the study, physical disabilities of the participants were not considered an exclusion criterion.

Participants received an introductory presentation by the researcher, informing them on the use and goals of the PERSSILAA app and game as well as on the study setup and aims of the research. People participated in pairs, and participants were invited to access the game and perform specific tasks or actions (divided over sets of 5 sessions to avoid knowledge saturation [42]) while speaking their minds and discussing with each other. The underlying exercises, as offered by the PERSSILAA app, were simulated to enable a walkthrough of the entire game in approximately an hour. Using screen capturing software, the actions and speech of the participants were recorded. After the session, participants were invited to fill out a questionnaire in a second room on separate PCs.

The questionnaire assessed demographics and information on current and past gaming behavior and experience with the use of devices by the end users. We measured the opinion of the participants using the Unified Theory of Acceptance and Use of Technology questionnaire [43] for performance and effort expectancy (76 items rated on a 7-point VAS). The formulation of the questions was adapted from the original to the technology used in this particular situation. The expected use of the game was measured by presenting a hypothetical use situation. In addition to the video and audio recordings, the questionnaire assessed the usability of the game in general and of specific aspects of the game. Scores on these aspects (overall appreciation; ease of use; performance; and effort expectancy, including expected effectivity versus motivation from the game, fun factor, and intended future use) were calculated. The preferences for game content of the end users were measured by means of 2 times 51 propositions on the content present in the game (5-point Likert scales).

##### Session 3

The third and last evaluation session used the same methods as session 2. However, the game was presented to the users in a stand-alone version that could be used in the participants’ home situation and was not played in couples but individually. No audio or video recordings were made. The questionnaire was filled out through a web-based form.

### Ethical Considerations

The user evaluations presented in this paper were part of the PERSSILAA project, sponsored by the EU (FP7-ICT- 2013-10) and the MAGGY (Mobile Activity Game for Elderly) project, sponsored by the Netherlands Organization for Scientific Research (NWO), Creative Industry Program (314-99-002). All participants provided their informed consent.

## Results

### Phase 1: End-User Research

#### Characteristics and Limitations

The target group was older adults aged between 65 years and 75 years with sufficient computer literacy to independently use mobile devices or a PC and an interest in digital games. Of the older adults questioned (N=136; mean age 69, SD 2.84 y), 77.2% (105/136) indicated that they play games, of which 75.2% (79/105) played at least once a week or even daily. Of frequent players, 73% indicated they played digital games (57/79, 72%, or 57/136, 41.9% of all participants), followed by board games or other conventional games. Computers and laptops (80/105, 76.2%) and smart devices (63/105, 60%) were the most popular platforms for playing. Two-thirds of the total number of participants (93/136, 68.4%; including solely board or card game players) chose playing together (cooperative or competitive) over playing alone. Only a few participants (12/136, 8.8%) indicated that they played online social games, of which Wordfeud (Håkon Bertheussen) was mentioned most often, or they were interested in doing so in the near future. The older adults preferred to play games at home rather than elsewhere or on the go (122/136, 89.7%).

Participants’ favorite digital games (Multimedia Appendix 1) were thematically categorized into card games (eg, Solitaire, FreeCell, and Spider), word games (Wordfeud, Ruzzle, and crosswords), digital versions of other conventional games (mahjong, chess, cryptograms, quizzes, and bingo), and modern online games (Candy Crush, Bubble Shooter, and search and find games). Interest in more modern games was relatively small, 13.2% (18/136) of all answers, but present. Participants played digital versions of conventional games, of which relevant examples were Scrabble and Rummikub. A diversity of card games and puzzle games were mentioned most often. Of all participants, 74.3% (101/136) indicated that they enjoyed trying out games they did not know. In interviews, many participants (24/136, 17.6%) expressed the belief that modern games are suitable only for younger generations or their grandchildren, but not for them.

#### Game Preferences

**Figure 4:**
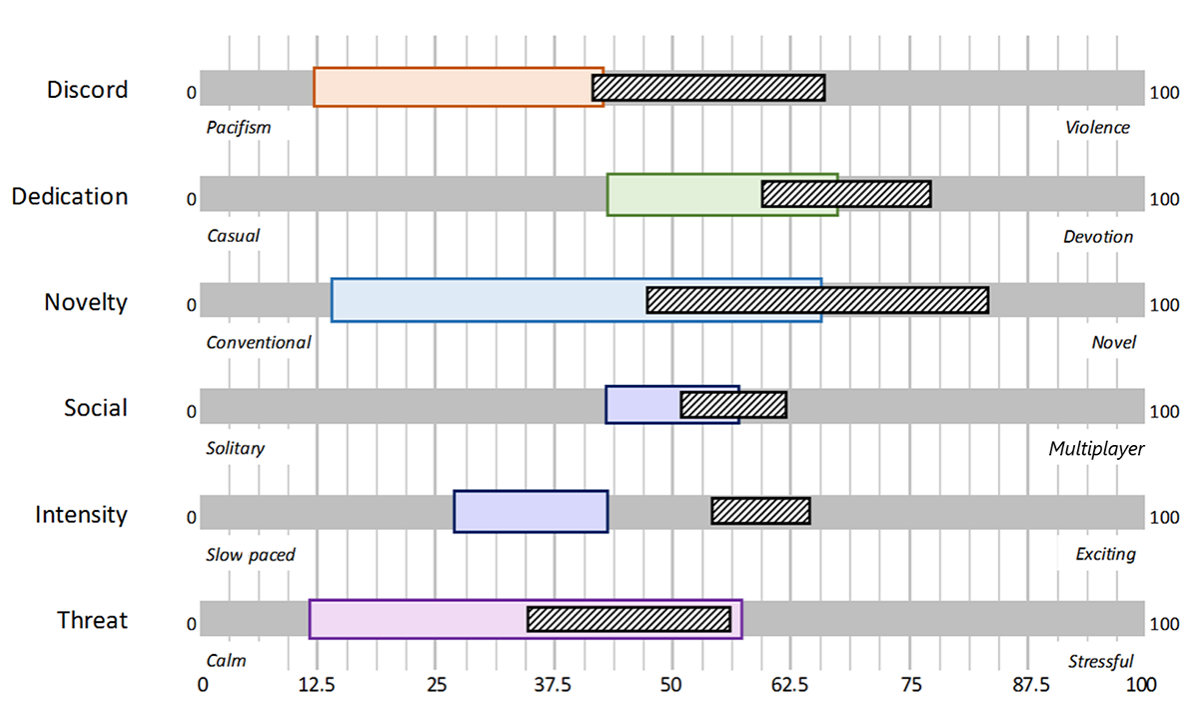
User profile (perception: taller solid bars and appreciation: shorter hatched bars).

The preferences of the older adults after playing new games or using a gamified app through questionnaires were assessed. Results of study 1 were presented earlier by de Vette et al [39]. In study 2, the scores on perception and appreciation of game content were mapped onto the classification, resulting in a user profile (Figure 4). The scores for the user’s perception of the game content are shown in colored, taller bars. This is the “game profile,” from the viewpoint of the older adult. The shorter, hatched bars indicate the user’s appreciation of this game’s content. Each domain is described by 2 extremes; for example, intensity is described by content that ranges from slow paced to exciting.

The profile illustrates the following findings: from the overlap in scores of the content of the game according to participants (“perception”) and the preferred game content for a satisfying gaming experience (“appreciation”), we noticed that for several participants, the game was insufficiently competitive and did not demand enough devotion. This included the challenge, effort needed to play, and learning involved. The game was considered conventional to moderately novel, including variation and aesthetics, which may not have been sufficient to engage the user. Participants rated the social component present in the game as neutral, as both solo and multiplayer options were integrated, while slightly preferring a multiplayer over a solitary game. The game was not sufficiently exciting for the participants, and the intensity of the game was rated below preferred. The contents of the game in the domain of “threat” were appreciated as they were, from which we conclude that the game was not too stressful or frustrating. As discord (violence) was not an apparent game feature, the score on this domain remains inconclusive at this point for use in PERSSILAA.

The additional interviews (for which we refer to the original article) give deeper insight into the abovementioned scores on the classification. First, most strikingly, participants were open to modern games and preferred less conventional game content than indicated in the earlier questionnaires. One of the games from study 1, Monument Valley, was particularly appreciated and played for many hours, even by those who were skeptical about (modern) video games beforehand. Participants indicated that playing video games had changed their attitude toward modern video games. Moreover, in study 2, the game concept of the eHealth app was not appreciated at all. The game was considered not novel enough, and the conventional game concept of crosswords was not found attractive. The game did not have enough variation to maintain engagement. Participants indicated the game was too similar to the games they already knew, and the gamified app therefore did not fulfill a need.

In the off-the-shelf games and the gamified app, problem-solving, intellect, and thinking were found to be particularly motivating. The puzzle and brain training aspects of the gamified app were appreciated by most users, despite being redundant among their current gaming behaviors. Aesthetics in the game, such as good graphics and attractive artwork, were appreciated. Other motivating game characteristics related to novelty are variation, curiosity, and discovery. Participants indicated that they thought a larger selection of games would contribute to their motivation to use the gamified app.

While the older adults had an interest in social gameplay in conventional games (board games and cards), they indicated that digital games were mostly enjoyed alone*.* In the interviews from studies 1 and 2, after the use of games or a gamified app, participants indicated that they found it enjoyable to withdraw and relax with a video game on their own. The participants indicated that they were competitive, but this aspect was not looked for in social contact through games. In the gamified app, 2 social playing modes were included (competitive and cooperative), but neither option was used by the participants. Participants indicated that they did not feel the need to share gameplay, the progression in the game, or their activity behavior with other people.

We observed that the older adults had a low tolerance for frustration and negativism. Game content that creates feelings of unfairness or lack of control should be avoided. Offering additional hints, cheats, or help may reduce feelings of frustration or incompetence, according to the users. Participants indicated feeling underestimated by presenting game content that was superficial and easy. A childish or silly theme would, however, not be appreciated, either. Participants indicated that fast-paced gameplay, demanding a high level of physical agility or reaction speed, was disliked in general. All participants indicated their strong aversion to violence.

Participants enjoyed being challenged or to challenge themselves, achieve goals, progress, and develop skills. Participants made use of statistics in the gamified app to challenge and improve themselves. Clear goals and trackable progression were mentioned as contributing to a positive experience in both studies. In the gamified app, the link between real-world activity and gameplay was not always clear, which was found demotivating. Trial and error in general was not an approach most older adults wished to take in games. Participants indicated that it was important that games can be paused and continued at any point and that controls that were simple and intuitive and goals that were clear at all times contributed to a positive experience.

#### Recommendations for Engaging Game Content

From the abovementioned results, we deduced a set of guidelines for game design for older adults. We aim to create a game that includes the characteristics shown in Textbox 1.

**Design characteristics.**

**Characteristics**

- Design for moderate to high novelty
  Novel game concept, offering variation, renewable content, enabling exploration, and triggering curiosity
  Attractive aesthetics and artwork, storyline, and graphics
  Focused on problem-solving, logical reasoning, thinking, and puzzles
  Provide clear rules and objectives.
- Design for moderate to high dedication
  Create challenging gameplay, enabling achievement, learning, and mastery
  Offer content for devoted gameplay, such as challenges and objectives with increasing difficulty
  High dedication should not be compulsory to play the game at all times; add free, casual gameplay elements and low-difficulty levels as well for balance
- Design for low discord and threat
  Neutral content; a relaxed and cheerful atmosphere; and not violent, overly friendly, or cute
  Avoiding content that triggers negative emotions such as stress, tension, a disturbing setting, frustration, anxiety, and unfairness
  Remove any possibility of failure and avoid negative feedback
- Design for moderate intensity
  A suitable game intensity is moderately paced, avoiding exerting game elements
  Demanding the focus of the user to complete objectives should be moderate or alternating, for example, multitasking at high speed should be avoided
- Design for low social interaction
  As our results did not strongly indicate the added value of a social game component, the initial version of Personalised ICT Supported Services for Independent Living and Active Ageing (PERSSILAA) will be focused on the solo player.

While we consider the available usability requirements for game interface design for older adults [44-48], we regard the following game-related usability specifications:

- Goals must always be clear and progress trackable as well as linked to those of the underlying health care app.
- Complex movements and controls should be avoided; game controls should be basic and intuitive.
- Completing game objectives may never relate solely on the agility of the player.
- The game should be accessible; simple game mechanisms are preferable over more complex ones.

### Phase 2: Conceptualization

#### System and App Architecture

The result of the eHealth app development is the PERSSILAA platform. The web-based self-management platform offers a monitoring and training program that supports both acquiring and maintaining a healthy lifestyle. This web-based service is particularly intended for people in a so-called prefrail state who show first signs of decline but are not yet in need of professional care. However, it is accessible to several users: the older adult or patient, the caregiver, and the health care professional. People with proper levels of functioning are, however, also encouraged to use the service, as training can prevent or delay becoming vulnerable to age-related health decline.

**Figure 5:**
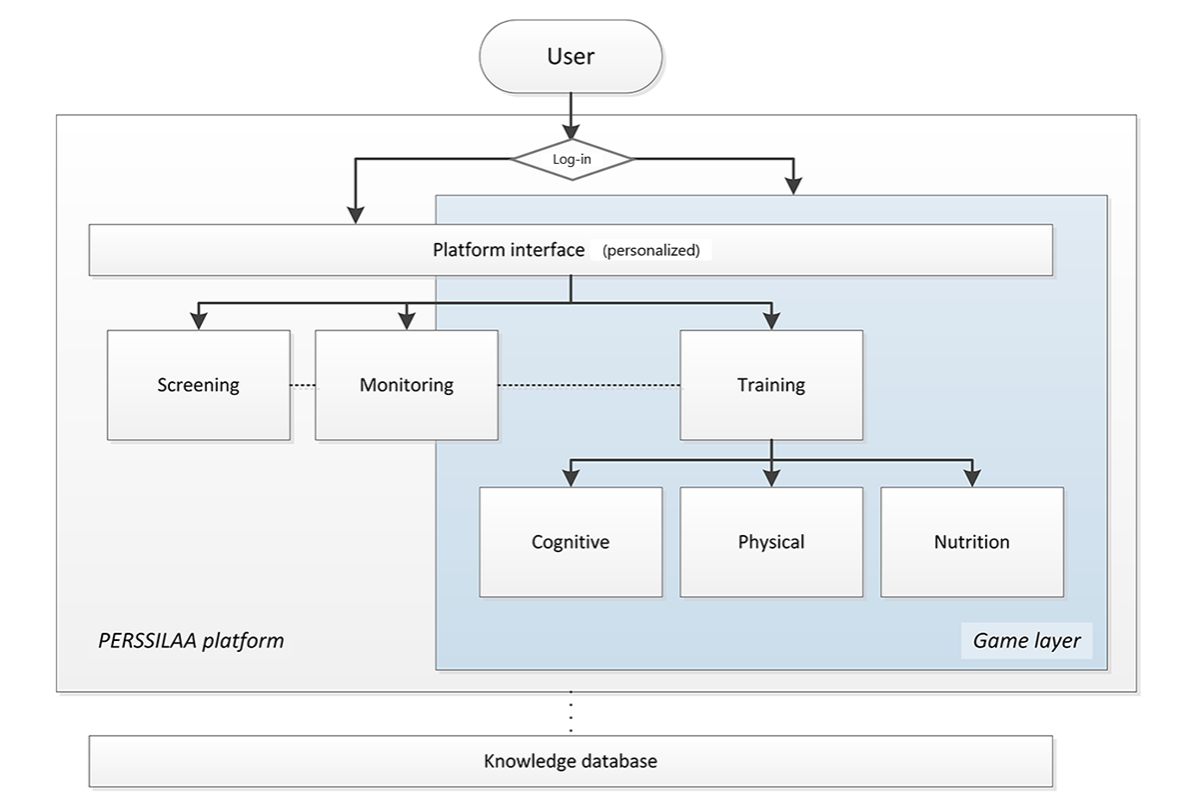
The Personalised Information and Communication Technology (ICT) Supported Services for Independent Living and Active Ageing (PERSSILAA) system for older adult end users. It has 3 functionalities: data-gathering questionnaires (screening); data on interaction with monitoring devices (monitoring) and training modules (training); and physical, cognitive, and nutritional well-being.

The functionalities of the platform for older adults are visualized in Figure 5. First, the user logs in and is given the autonomy to always choose between the “standard” and “game-based” versions of the platform. From within the game, the personalized (nongamified) platform interface, or “start screen,” can be recalled. The collection of data that occurs through the screening process module, through questionnaires, and the network of sensing devices forming the monitoring module, such as a step counter and digital scale, is out of scope for this paper. The functionality of the game-based version is limited to the training modules and a representation of selected monitoring data.

We define four versions with increasing added functionality of the standard version into the game-based version:

1. Prototype—due at the end of the refinement phase (phase 4 of the gamified design process). Functionalities from the original app to include the development of the prototype: training modules and basic monitoring.
2. First follow-up—due at the end of the implementation phase. Functionalities are those of the full platform: training, monitoring, and screening.
3. Second follow-up—due at the maintenance phase. Planned functionalities for the eHealth app that should be part of the gamified version in the future (wearables, smart devices, and digitizing of the intake process). Extension of game design such as an interactive social platform or extension of already implemented functions.
4. Future versions—opportunities for future game design functionalities, unrelated to or derived from the underlying app.

#### Long-Term Engagement Strategy

Interviews with developing partners resulted in (1) a list of activities for each particular health care domain that were identified as relevant indicators of user performance and (2) the assignment of fictive GUs to the identified performance indicators. An excerpt of the performance indicators and their respective GUs for the physical module is shown in Table 1. The physical module provides exercises for strength, endurance, and mobility using diverse monitoring and feedback methods on daily activity patterns.

**Table 1:** Performance indicators of physical training.

Indicators		GU^a^	Exercises, n	
**Warming up (max+6 GU)**				4
	Commencing	+1		
	Per finished exercise	+1		
**Training session (max+11 GU)**				9
	Per finished exercise	+1		
	Finishing training (limit: 5 skipped exercises)	+2		
**Cooling down (max+6 GU)**				4
	Commencing	+1		
	Per finished exercise	+1		
	Finishing	+1		
Full session		+5	—^b^	
Answering questions		+1	—	
Regularity of training		+5 (1.05/wk)	For example, 3 times weekly	

^a^GU: game unit.

^b^Not available.

The interviews resulted in notes of caution regarding possible misuse. Encouraging practice while experiencing pain must always be avoided. Skipping exercises or terminating the training must not be discouraged, and a limit of skipped exercises to complete a session may be set. Stimulating the user to practice exercises that are below their current performance level, to generate quick rewards in the game, should be avoided. Cheating the system just to gain access to new game content should be prevented as much as possible but cannot be avoided in each case.

### Phase 3: Game Design

#### General Game Concept

The general game concept is interactive, scalable, and expandable. To the user, this gaming environment is an alternative interface that, if desired, fully replaces the original app. The gaming environment is scalable in terms of adaptability to fit the user-specific needs for access and restrictions to underlying platform functionalities. It is built for expansion with new game content to extend the time that the game is interesting to play, in such a way that changes to the original platform do not impair the functionality of the game even during use by the end user.

Following the results of phase 1, various gaming concepts were explored. A theme was created that allowed sufficient variation and exploration for the user. This gives clear rules and boundaries and can be combined with an aspect of logical reasoning and a puzzle. The chosen concept is a map-based and story-driven game to stimulate intrinsic motivation driven by curiosity and achievement. Ideas that emerged from the sessions were mini games on different map locations, including activities related to the themes of the modules, and a showcase of trophy items to gather over time.

#### Quantifying Performance

**Figure 6:**
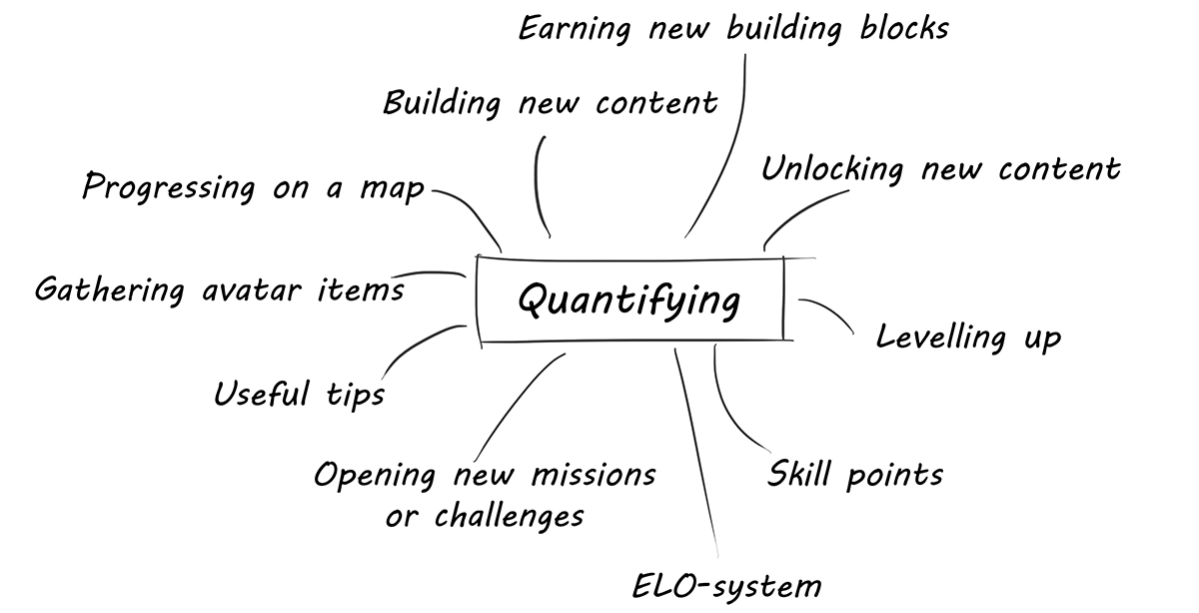
Brainstorming notes from the sessions on possible solutions for the representation of performance. ELO: expected likelihood of outcome.

From the general game concept, a suitable representation in the game of the performance of the user on the underlying training modules was created. Figure 6 shows one of the brainstorming notes. The representation of performance, through the GUs, must receive a suitable, unobtrusive antagonist in the game environment.

The storyline in the game is the main mechanism to provide feedback and motivation for the activities in the training modules. It was planned to integrate game content for a total playing time span of 12 weeks, as this reflects the optimal use time of the original app’s training and monitoring goals. Through user performance, the environment evolves and new areas to explore are unlocked. These areas contain gameful activities (eg, mini games) that can be played freely, which are again rewarded with a part of the storyline of the game.

#### Core Gameplay Features

**Figure 7:**
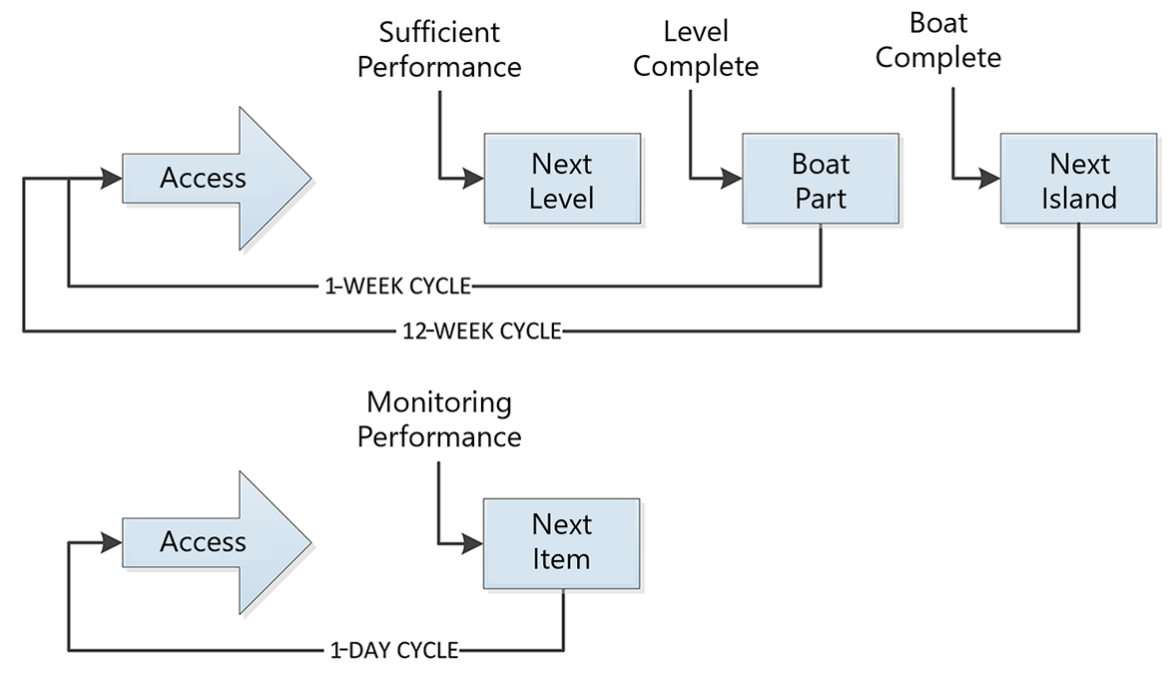
Flowchart of the game progress of Stranded!

The game was titled Stranded! (translated from Dutch “Aangespoeld!”). The high-level narrative brings the player to an adventurer in late Victorian times who is shipwrecked on an unknown island in a storm. The parts of the boat that have been scattered all over the island must be retrieved to build a new boat to reach home before the volcano erupts. Every next level is opened after passing a personalized threshold of performance on the platform defined by GUs. By completing each level, which will have a puzzle character, a part of the boat can be gained. Eventually, when the user has completed the boat, the next island can be made available to explore (Figure 7).

The game environment is built up from several scenes. Major locations are as follows:

- An overview screen, or map, of the first island (bird’s-eye view)
- A 2-part landing page or home screen, similar to the portal, gives access to the modules as well as the pier where the lifeboat is constructed
- A total of 12 in-level screens, such as mini games and representations of the modules for monitoring and nutritional advice

The player starts at the home screen in each new session. When the game is started for the first time, an opening animation will show that introduces the backstory. From here, the training modules can be accessed directly through small wooden cabins. The scalable aspect of the game layer is realized through these wooden cabins, which can be added or removed to suit the training advice of the end user. The screen pans if the player walks to the left of the beach, showing another cabin that is connected to the monitoring module as well as the boat pier. From the beach, the player can move to the island in the bird’s-eye view. This map of the island will, in time, reveal new locations in which mini games with their own levels and graphic concepts can be played. The first version of the game layer is expandable, with more map areas and levels in mini games. The monitoring layer interacts with the game environment through the arrival of messages in a bottle, which contains items that can be stored in a trophy hut, reflecting the overall timeline and monitoring achievements of the user. A social component for a future version could be added by adding the function of taking snapshots of scenes and environments that can be shared with others. The cycle of 12 weeks can be prolonged by extending the storyline to a second main location or island.

#### Concept Art

Concept art covers the visual and auditory outline of the game, including graphics, animations, and sound design. Results in this section are style mood boards and interface mock-ups, graphic-level (mini game) themes, character drawings, animation storyboards, game music compositions, and sound effects. A summary of concept development is given in Multimedia Appendix 2. Intro and outro animations can be seen in Multimedia Appendix 3.

### Phase 4: Refinement

#### Prototypes

**Figure 8:**
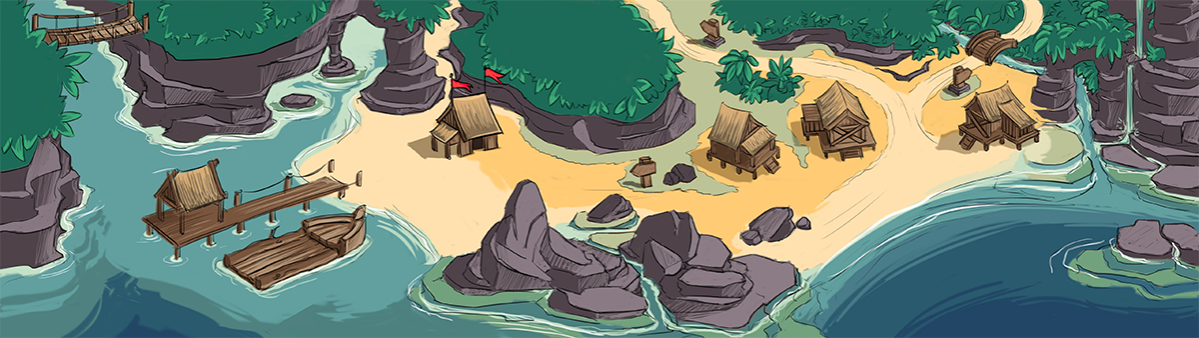
Final graphic design of the home screen.

The final prototype was created through an iterative prototyping and evaluation process. The initial version was the result of the research described in phase 1 as well as rigorous playtesting with the development team and researchers. In each playtest, the full game was played, and all functionality was checked. The functionality of the game was constructed and tested using simple visuals that were exchanged for mock-up images and, when finished, a final graphic design (Figure 8). Multimedia Appendix 4 shows the screenshots of these development cycles. Three more evaluation sessions with end users led to the refined final version of the prototype. An information point providing short explanations of items and functionalities on screen (an i-icon in the corner of each screen) was created. Finally, information was gathered on refining future versions (in the maintenance phase) as well as preliminary implementations for future functionalities.

The main character is a female explorer named Sophie, a likeable character that fits the storyline well, rather than a character that resembles the players themselves. In the opening animation, we see Sophie on board a ship that is caught in a storm. She falls overboard when a sudden wave tilts the ship, and she washes up on the beach of a seemingly desolate island.

Every session starts with the choice of a classic or a gamified portal using a slider. The player starts on the right side of a panning scene showing a beach. The player can start exploring the area and access huts that are directly connected to the training modules. The user can access a crop field and cooking area (“moestuin,” to be paired with nutritional advice), and the rest of the island allows them to explore minigames (“naar het eiland”) or the left side of the beach where the boat is built (“boot”). Items that wash up on the beach in bottles and some interactive surprises (or “easter eggs”), which are crabs, were created. These crabs seem to be noninteractive, but after some trying, it appears that they can be caught. These bottles are the main feedback from the monitoring module. Some contain seeds that are earned through training and through monitoring sufficient exercise, which can be planted and grown in the crop field. Once harvested, they can be used to cook meals. The left hut is opened and leads to the physical training module. The other huts are constructed to be coupled with the nutritional and cognitive modules.

The island map displays 6 levels that can be played, which have been opened by completing training or exercises. After finishing each level, a piece of the lifeboat will be added to the construction. The image shows the progress after approximately 2 weeks of practicing and playing.

#### Evaluations

Table 2 presents a summary of the findings from the evaluation studies.

**Table 2:** Summary of the results of evaluation studies.

		Round 1	Round 2	Round 3
**Participants (n=35), n (%)**				
	Male	7 (54)	7 (58)	5 (50)
	Female	6 (46)	5 (42)	5 (50)
	Total	13 (100)	12 (100)	10 (100)
Of which regular players (weekly to daily), n		9	7	9
Age (y), mean		69	69	69
Overall appreciation for the game (%)		—^a^	73	74
Ease of use of the app (%)		—	73	73
Expected effectivity and motivation through the game (%)		—	61	63
Fun and pleasantness of using the app (%)		—	76	73
Intended future use (%)		—	64	67

**^a^**Not applicable.

From the log files of the first evaluation round, it was observed that 9 people explored the game without playing actively before returning to the use of the standard platform. Among the reasons given were computer issues (n=1), looking too complicated (n=1), and distracting from the original exercises (n=1). Two participants indicated that they had forgotten about the game and that they would like to receive another chance. The game was played in combination with the underlying physical activity training module by 4 participants (2 male and 2 female participants) for a longer amount of time (1-6 weeks, 1 person weekly, and 3 persons daily). Of these 4 participants, 3 (75%) were frequent players. The appreciation for the game ranged from mild to extremely enthusiastic. Two people indicated they were highly motivated to do the exercises because of the game. None of the participants were interested in playing the game together or sharing their progress. In the game, the storyline and the overall gameplay were the factors that appealed most to the participants, while the in-game explanations were found to be too limited. Two participants found the level of challenge in the mini games just right; 1 found it too difficult and 1 found it too easy. The controls of the game were not found to be sufficiently intuitive to use.

Using this information, pop-ups with short instructions or explanations on the current section of the game, which can always be recalled by clicking on the information icon in the corner of every screen, were implemented. In addition, the mini games were given a tutorial through a very easily solvable first level with detailed explanations. The controls of the main character were revised to make them easier to understand and activate by reducing moving around to just 1 click or tap on the screen.

In the second round of sessions, the game was played from beginning to end using instructions from the researcher. Participants indicated that the minigames (5/12, 42%) and gathering boat parts (4/12, 33%) were the game’s best features. The gamified platform (including the exercises) would be recommended to peers by 7 participants, and 8 participants would like to play the game again in the future. From the analyses of video (thinking aloud method) and screen captures, we observed that the explanatory text of the mini games was skipped by most participants. One of the minigame controls was not understood at all because of this. Participants said they did not want to take time to read the text or thought they would understand the games without the explanation. None of the participants returned to this information afterward but were inclined to give up without trying.

After this round of sessions, minor usability issues in the game were solved. The minigames had several unclear aspects that appeared to be caused by the explanations not being read. Therefore, the introduction to the new minigames was improved with shorter and more clear images. The minigame that could not be played by most participants was fully replaced by another game.

In the third and final round, the prototype game was used in the daily living environment for 6 weeks, again using simulated exercise. The scores that the questionnaire returned were very close to those of the prior session. Five people would recommend the gamified platform to peers; 5 people would recommend the underlying exercises to peers. One participant mentioned sometimes having difficulty starting the game, and 1 participant found that playing was fatiguing for the eyes (no playing time was mentioned). Six participants would like to continue using the game. Challenges and goals were appreciated by most people, and playing the mini games was again considered the most motivating aspect of the game. The crop field was appreciated the least. During the final evaluation round, no new usability issues emerged, and no further errors occurred that hindered the use of the web-based app.

**Figure 9:**
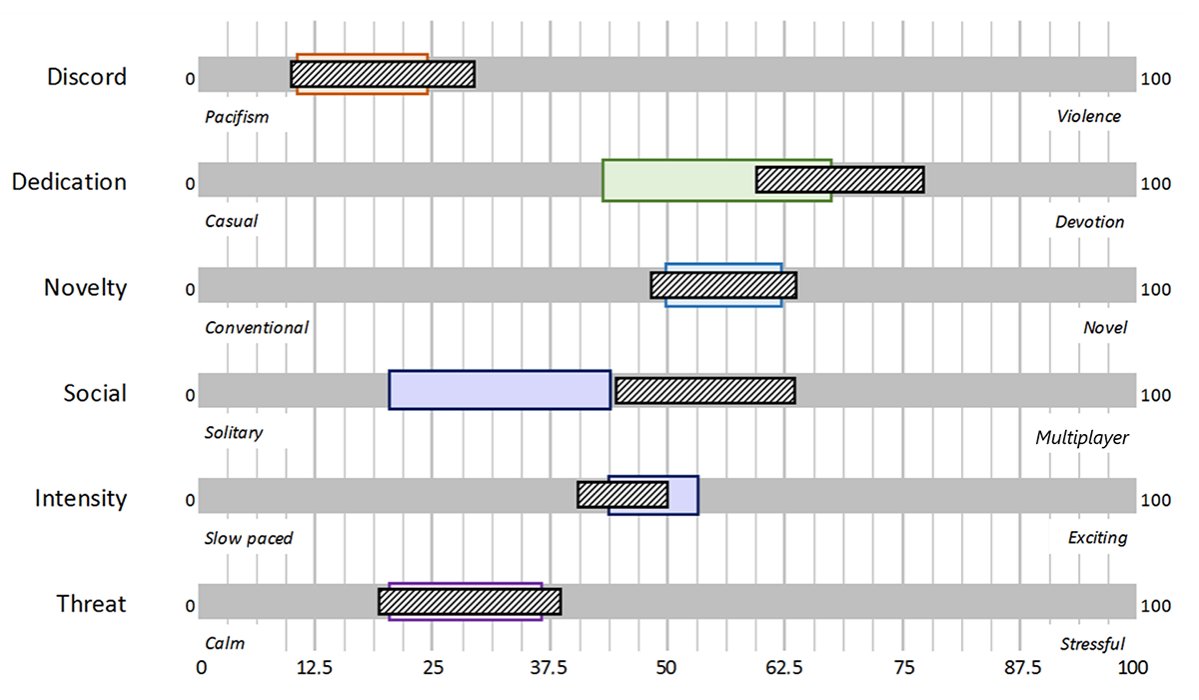
User profile (perception: taller solid bars and appreciation: shorter hatched bars).

As described earlier, the perception and appreciation of participants for different aspects of game content were measured in each of the evaluation rounds (26 participants returned complete results). Answers were given using a 5-point VAS and transposed to percentages before analysis. The 95% CIs were calculated and visualized in a graph (Figure 9). This figure helps to gain insight into how the game content is perceived by the user, related to the appreciation of the same content.

In our prestudies, we noticed a clear difference between the measured perception (or interpretation) of aspects of a game that was tested and the measured appreciation for these aspects by the user. In testing the PERSSILAA game, the scores of perceptions and appreciation did not show such large discrepancies. The difference between the 2 average values for each of the domains (as explained in the Phase 1: End-User Research section) was smaller than 4/100 for all domains except dedication and social, for which the differences were around 20/100. We, therefore, carefully assume that the guidelines we set in concluding the end-user research described in the Phase 1: End-User Research section have helped us create attractive game content for the computer-literate older adult users. As stated earlier, the social component deserves further exploration.

## Discussion

### Principal Findings

This study provided insight into the development of game-based eHealth in practice from beginning to end. We hope that this paper aids researchers and designers of future game-based apps. We introduced 4 phases in the process toward game-based eHealth: end-user research, conceptualization, creative design, and refinement. These phases can be integrated into general system development cycles (eg, [49,50]) or into specific frameworks for the development of eHealth apps [51]. Our case study demonstrated the application of the proposed game design process and resulted in a complete game-based eHealth app. Furthermore, user evaluations of the prototype app indicate that results from end-user research and consequential strategies for long-term engagement led to game content that is engaging to the older adult end user, supporting the value of our approach. We believe that by offering transparency in our approach to the design process and by providing practical examples, we contributed to opening the black box of game design that supports engagement in these apps.

### Game-Based Design Process: Limitations and Future Research

Our iterative, 4-phase method for game design gives prominence to the development of the actual game content within the overall eHealth app development. In a practical sense, however, such methods may also pose difficulties, as related work shows. For example, Hussain et al [52] state that priority conflicts arose from stakeholders less acquainted with agile approaches. In our case study, we recognized this issue and responded to it by prioritizing stakeholder influence in the design process in advance. This helped ensure that creative game design was given the necessary consideration and prevented it from being overshadowed by technical requirements or demands from the health care perspective. While satisfying each stakeholder, space was created for the creative process leading to the design of fun and attractive game content.

From applying the game design process in practice, we learned that engagement was subject to many prerequisites. For example, the app must not only have engaging content but also offer added value beyond the underlying app alone. Usability should not be a barrier, and the game must be well designed and well executed. However, end user characteristics were also influential. We found that an “ideal” end user for game-based eHealth is open to the technology, able to work with this technology without extensive instruction and support, and preferably enjoys games and play. Furthermore, eHealth can play a supporting role as this user intends to work on the health objectives offered [53]. Participants included in our studies were ideal users: able to use the devices well and open to games and technology for use in eHealth. Therefore, they stand as models for the older adults of the future rather than being representative of older adults at the time of writing (2018). The game-based PERSSILAA platform and design recommendations are therefore no guarantee for success when applied to situations with different (health care) objectives or population samples. Repetition of the game-based design process as presented can (and should) lead to different requirements for game design when applied elsewhere.

### Case Study: Limitations and Future Research

In the case study, the game-based version of the eHealth app was evaluated in a simulated setting (phase 4 of the game design process: refinement). The full rollout of the finalized product will occur after concluding the game-based design process in the implementation phase (Figure 1). The performance of the game environment in terms of motivation and engagement must be explored in its actual use situation in future studies. Pilot testing may lead to new insights affecting any of the prior development cycles. Follow-up research topics may include both evaluations of the developed game-based app in terms of user experience and satisfaction in real-world settings (including real exercises). In addition, exploration of future functionalities in terms of social interaction, user-generated content, and expansion of existing features for prolonged use times may contribute to sustaining engagement over time.

Results from phase 1 (end-user research investigating game preferences of end users and specifications from the use context of the envisioned app) indicated that the obvious gaming preferences of older adults based on current gaming behavior (eg, Scrabble and cards) may not lead to successful concepts for game-based eHealth. As mentioned in the Introduction section, we approached the investigation of potentially engaging content from the perspective of entertainment games and game design [36]. While these are not restricted by underlying “serious” goals and purely aim for a satisfying experience, future research should focus on gathering additional knowledge on how to determine preferences for game content among end users to be used in current and future game-based or gamified eHealth apps [40].

## Appendix

Multimedia Appendix 1 Participants’ current favorite games.

Multimedia Appendix 2 Overview of concept art.

Multimedia Appendix 3 Game animations (intro and outro).

Multimedia Appendix 4 Screenshots of the prototype phases.

## Abbreviations

GU: game unit
PERSSILAA: Personalised ICT Supported Services for Independent Living and Active Ageing
VAS: visual analog scale
