# Supplementary figures and images for: Developing Game-Based Design for eHealth in Practice: 4-Phase Game Design Process
Source: JMIR Form Res. 2024 Nov 8;8:e13723. doi: 10.2196/13723 (PMC11584551; doi:10.2196/13723)

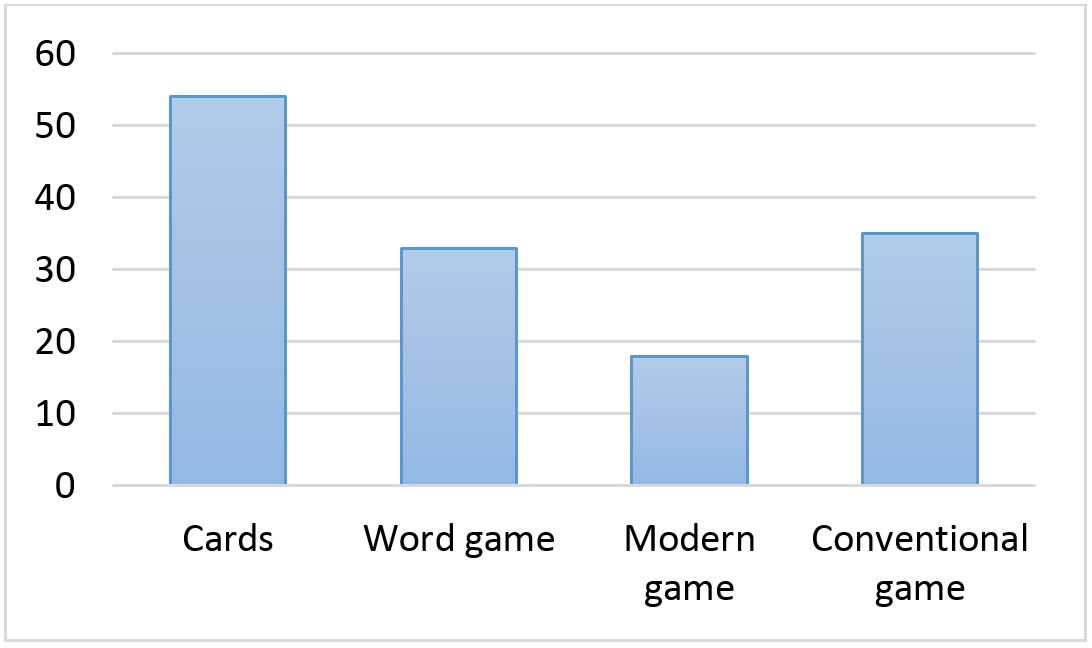

Supplement: Multimedia Appendix 1 [file formative_v8i1e13723_app1.png]

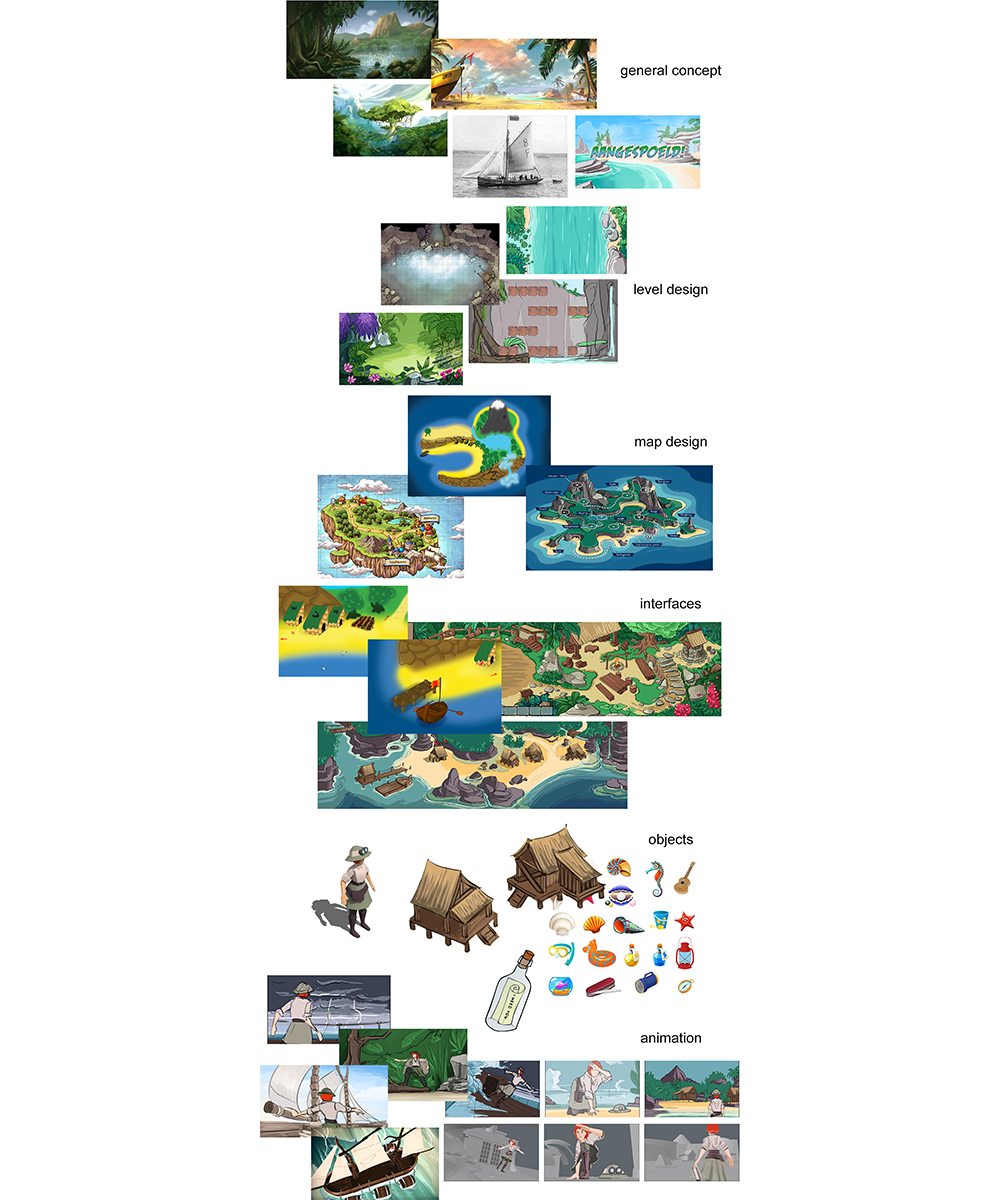

Supplement: Multimedia Appendix 2 [file formative_v8i1e13723_app2.png]

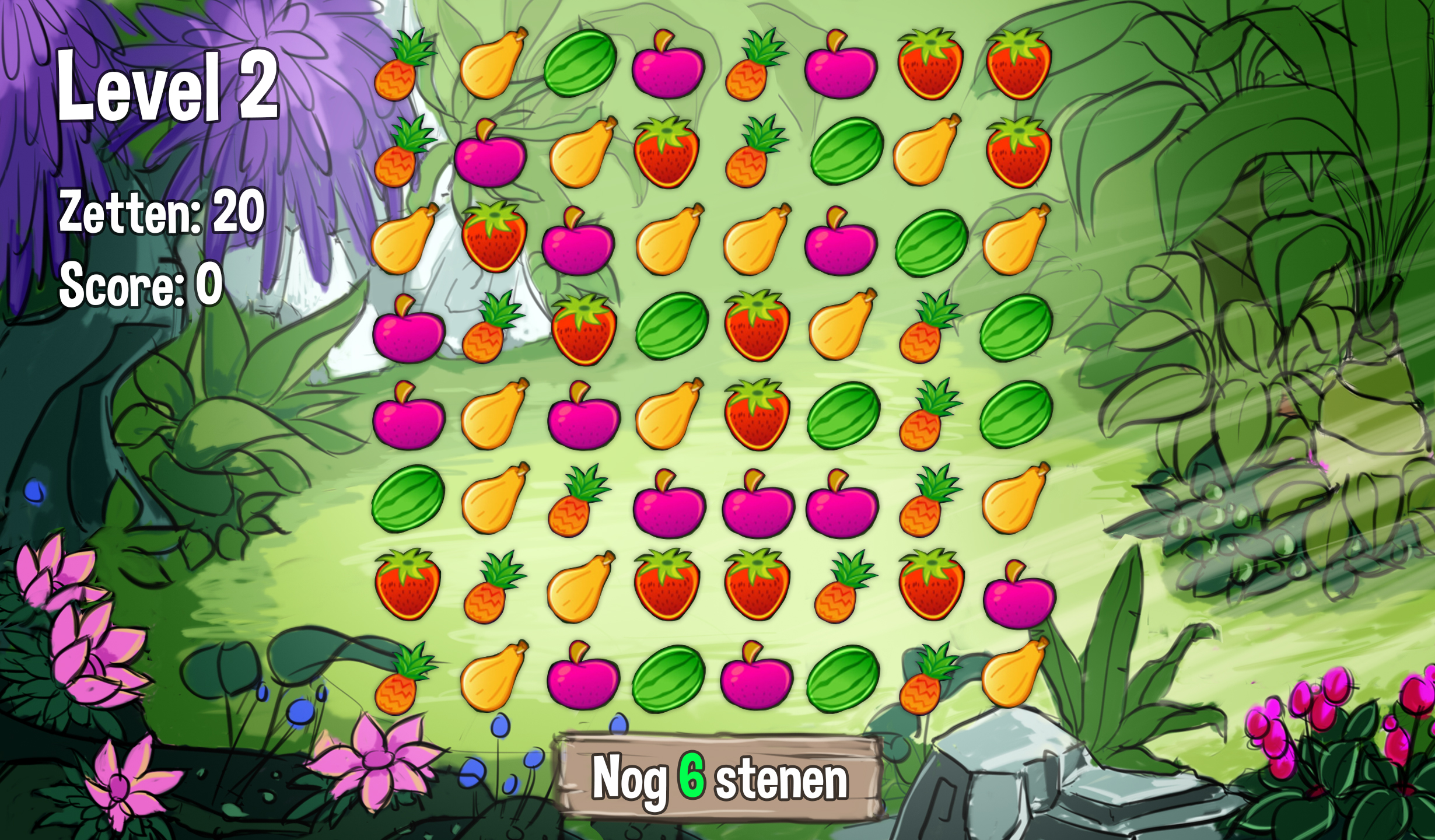

Supplement: Multimedia Appendix 4 [file formative_v8i1e13723_app4.zip › MMA2 - Prototype 3.5.jpg]

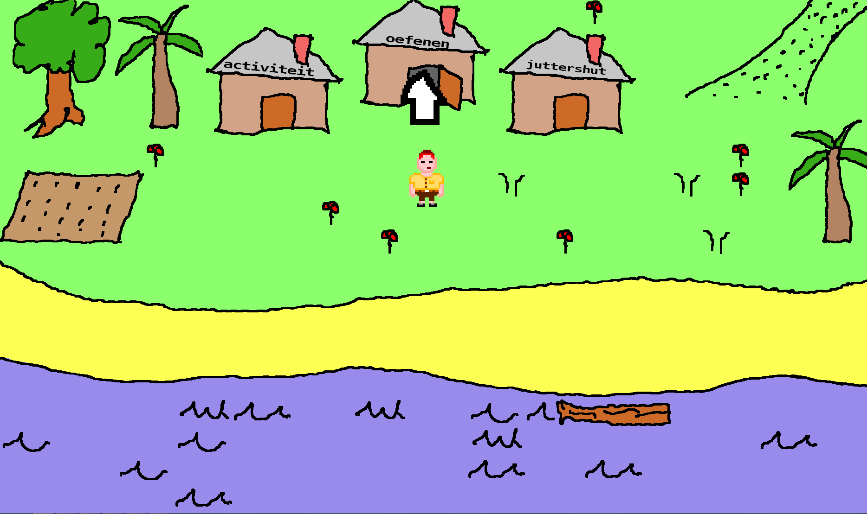

Supplement: Multimedia Appendix 4 [file formative_v8i1e13723_app4.zip › MMA2 - Prototype 1.1.png]

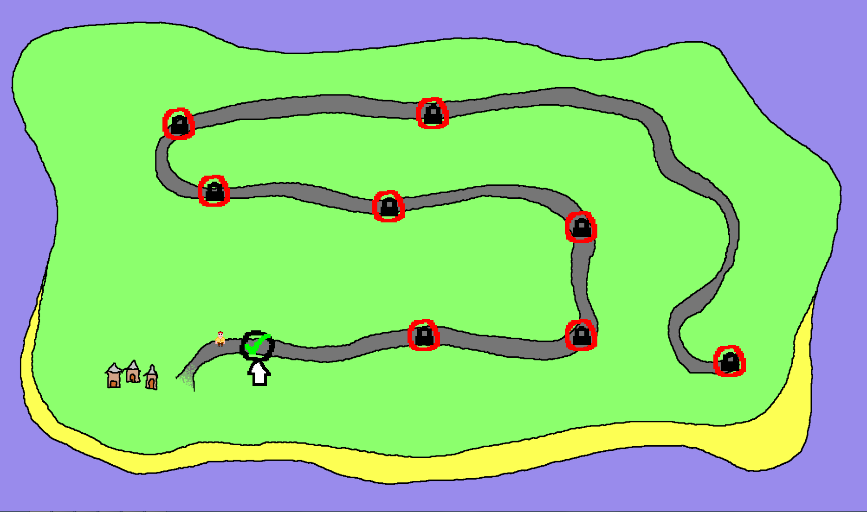

Supplement: Multimedia Appendix 4 [file formative_v8i1e13723_app4.zip › MMA2 - Prototype 1.2.png]

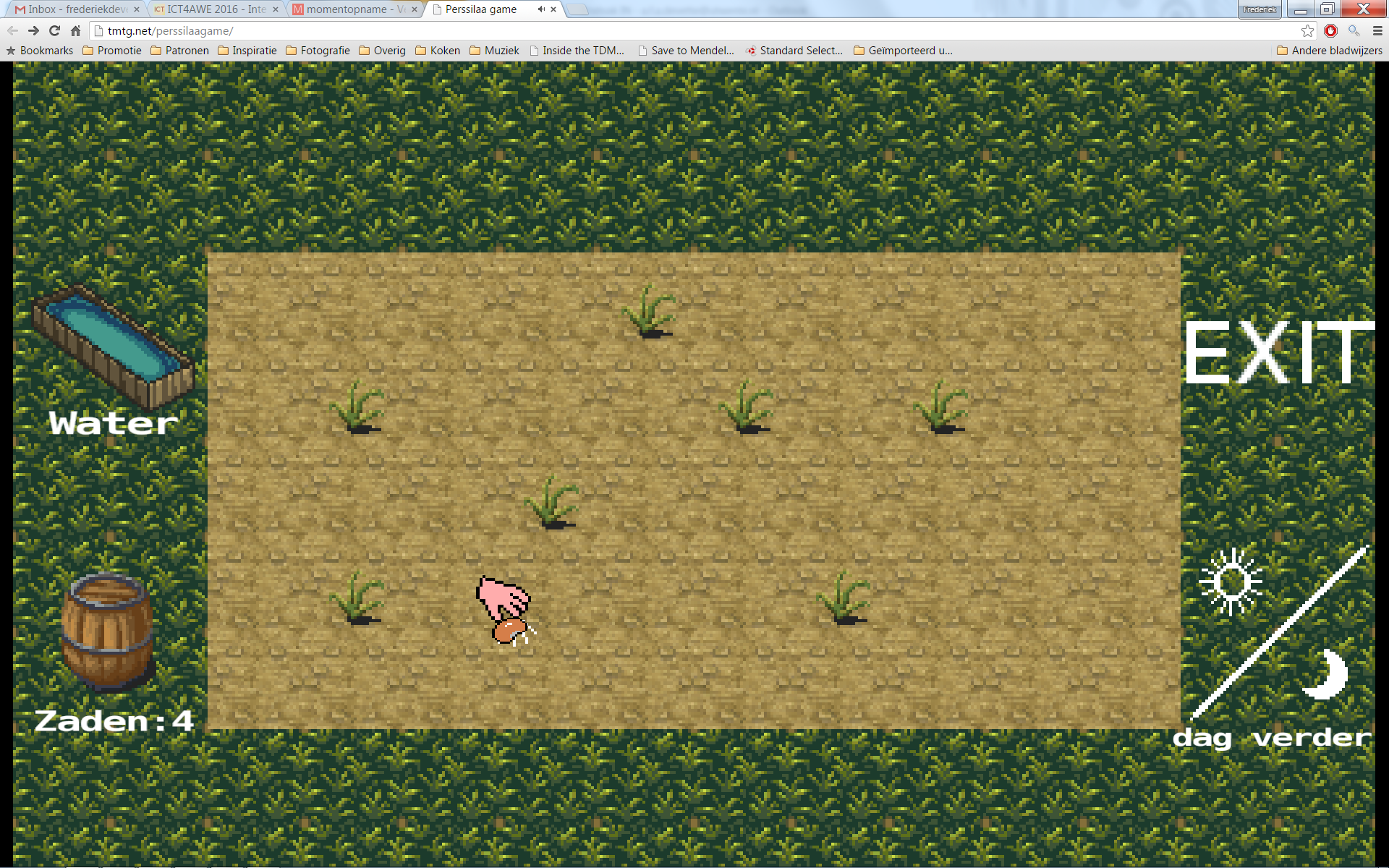

Supplement: Multimedia Appendix 4 [file formative_v8i1e13723_app4.zip › MMA2 - Prototype 1.3.png]

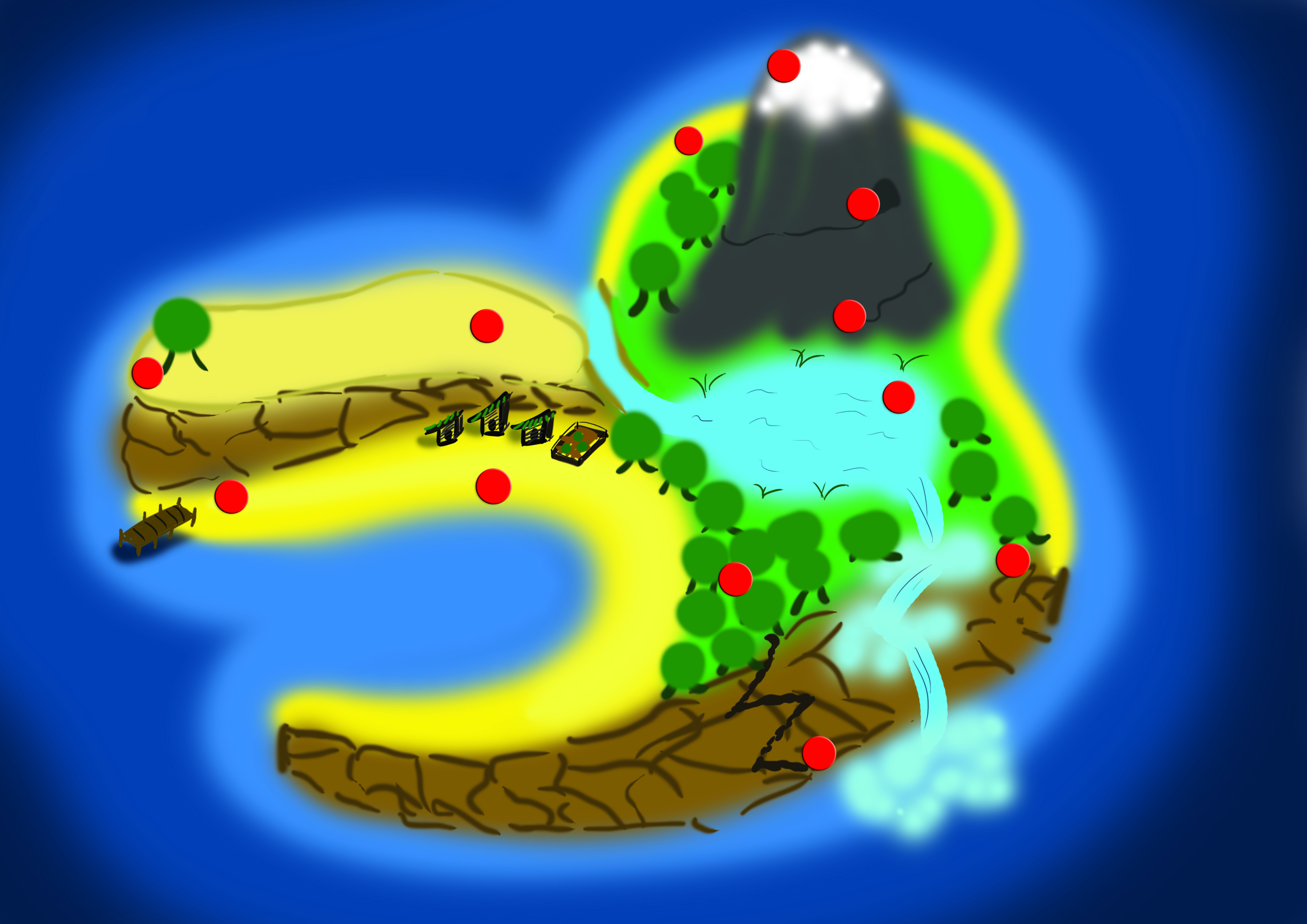

Supplement: Multimedia Appendix 4 [file formative_v8i1e13723_app4.zip › MMA2 - Prototype 2.1.JPG]

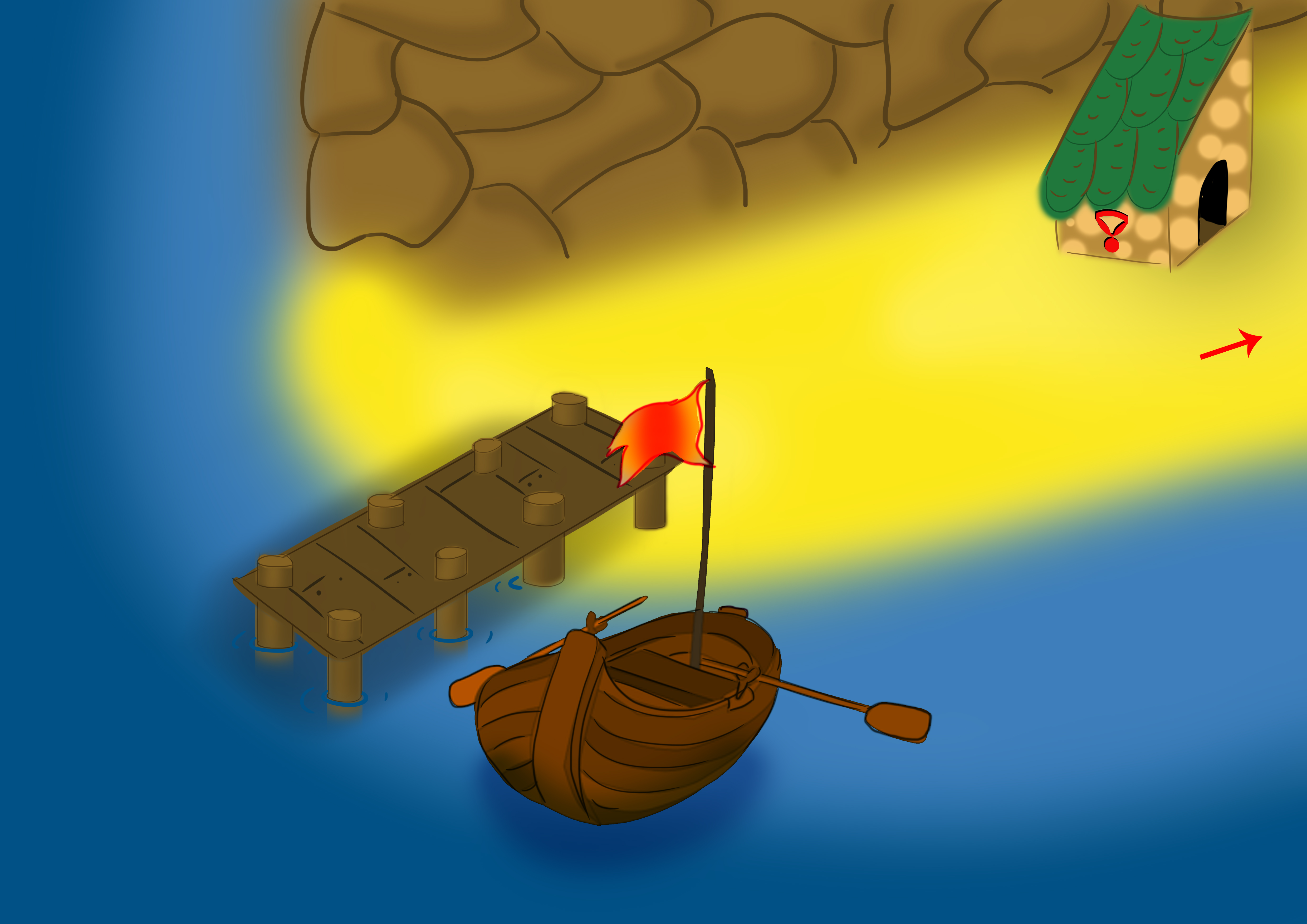

Supplement: Multimedia Appendix 4 [file formative_v8i1e13723_app4.zip › MMA2 - Prototype 2.2.jpg]

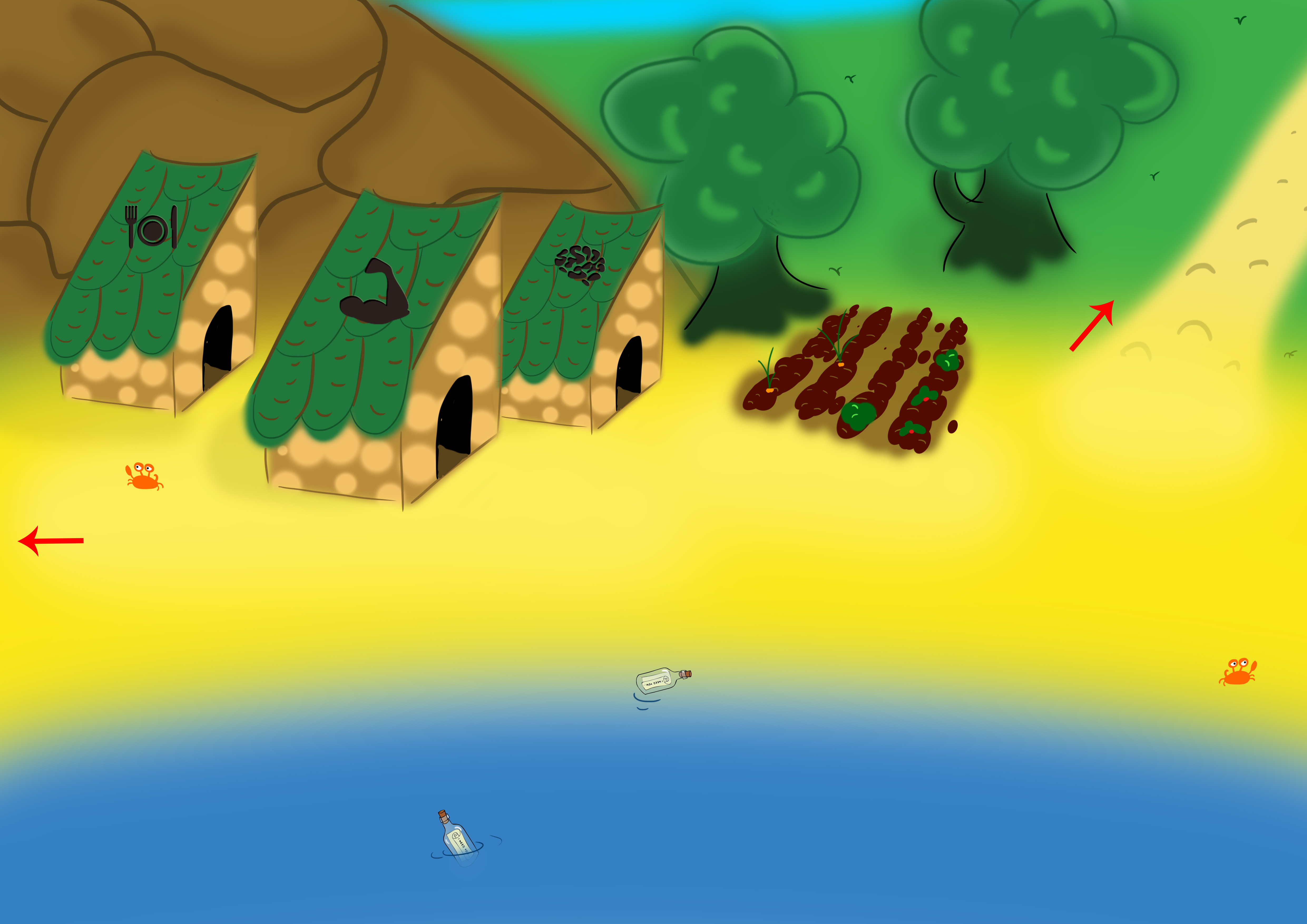

Supplement: Multimedia Appendix 4 [file formative_v8i1e13723_app4.zip › MMA2 - Prototype 2.3.jpg]

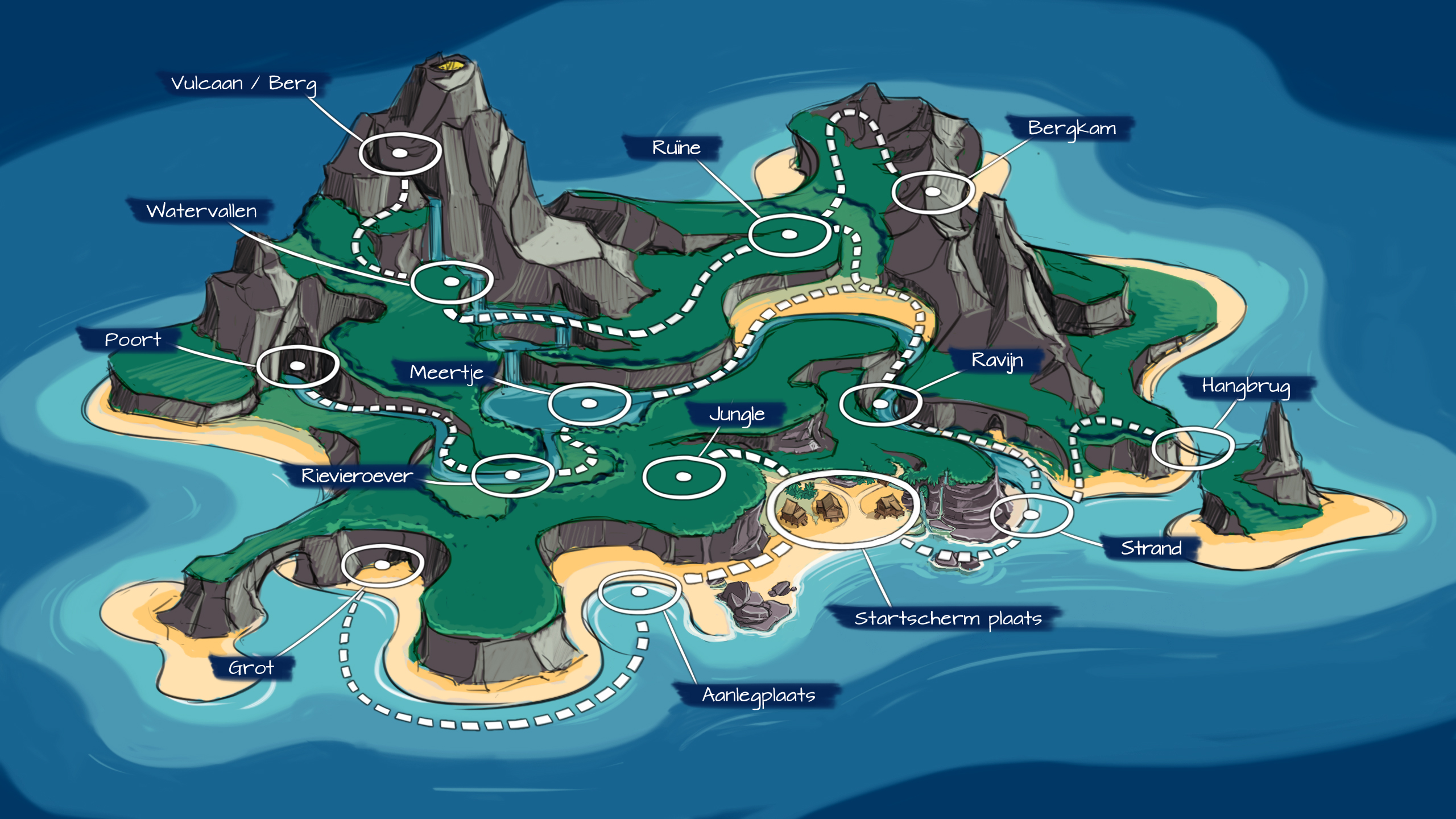

Supplement: Multimedia Appendix 4 [file formative_v8i1e13723_app4.zip › MMA2 - Prototype 3.1.jpg]

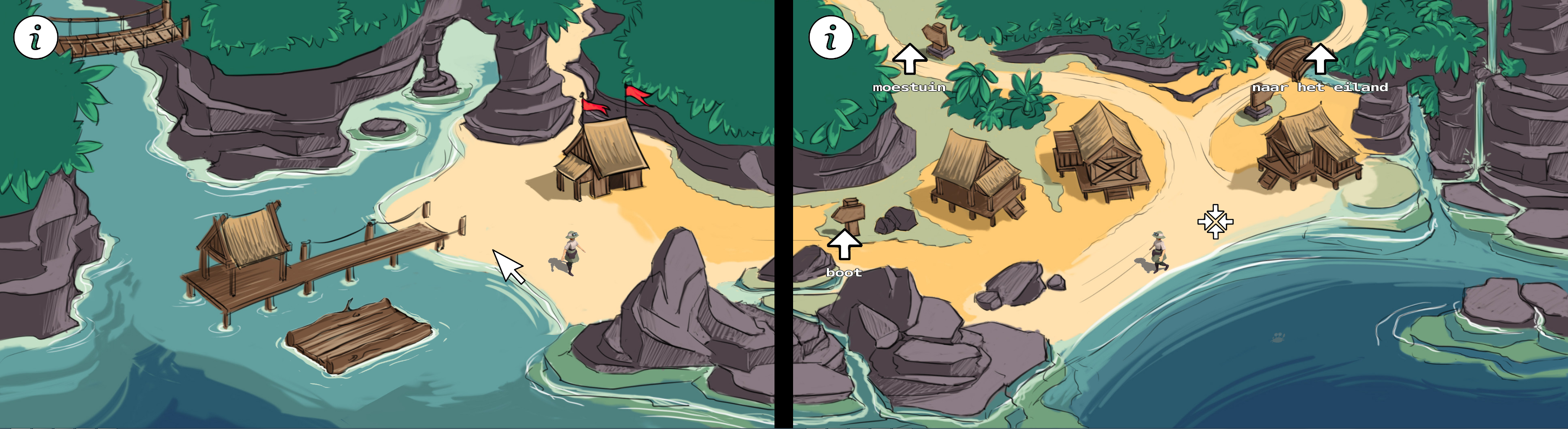

Supplement: Multimedia Appendix 4 [file formative_v8i1e13723_app4.zip › MMA2 - Prototype 3.2.png]

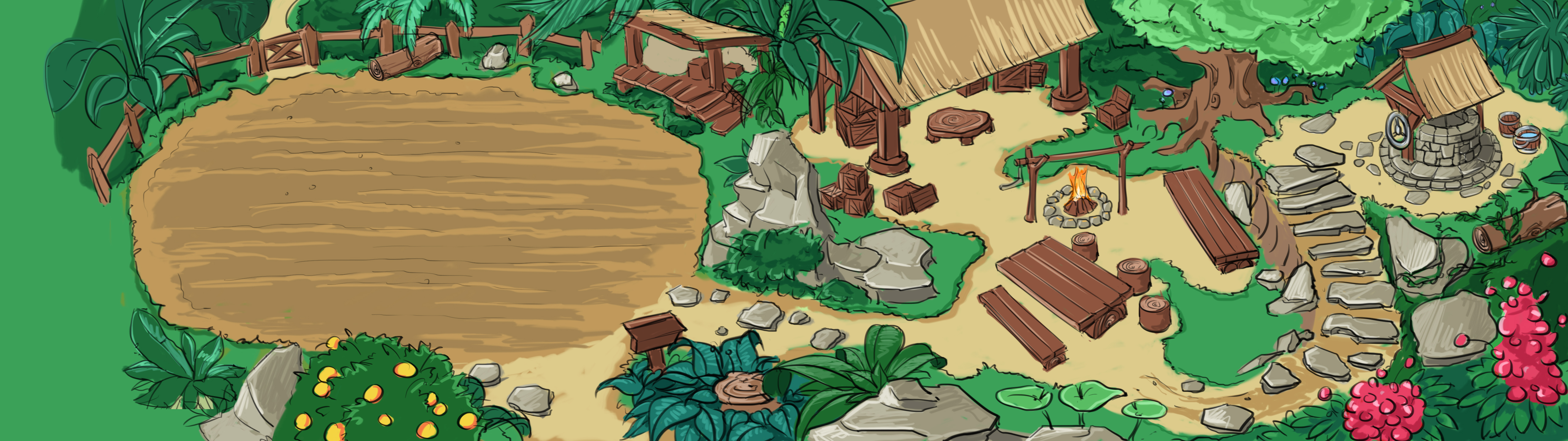

Supplement: Multimedia Appendix 4 [file formative_v8i1e13723_app4.zip › MMA2 - Prototype 3.3.jpg]

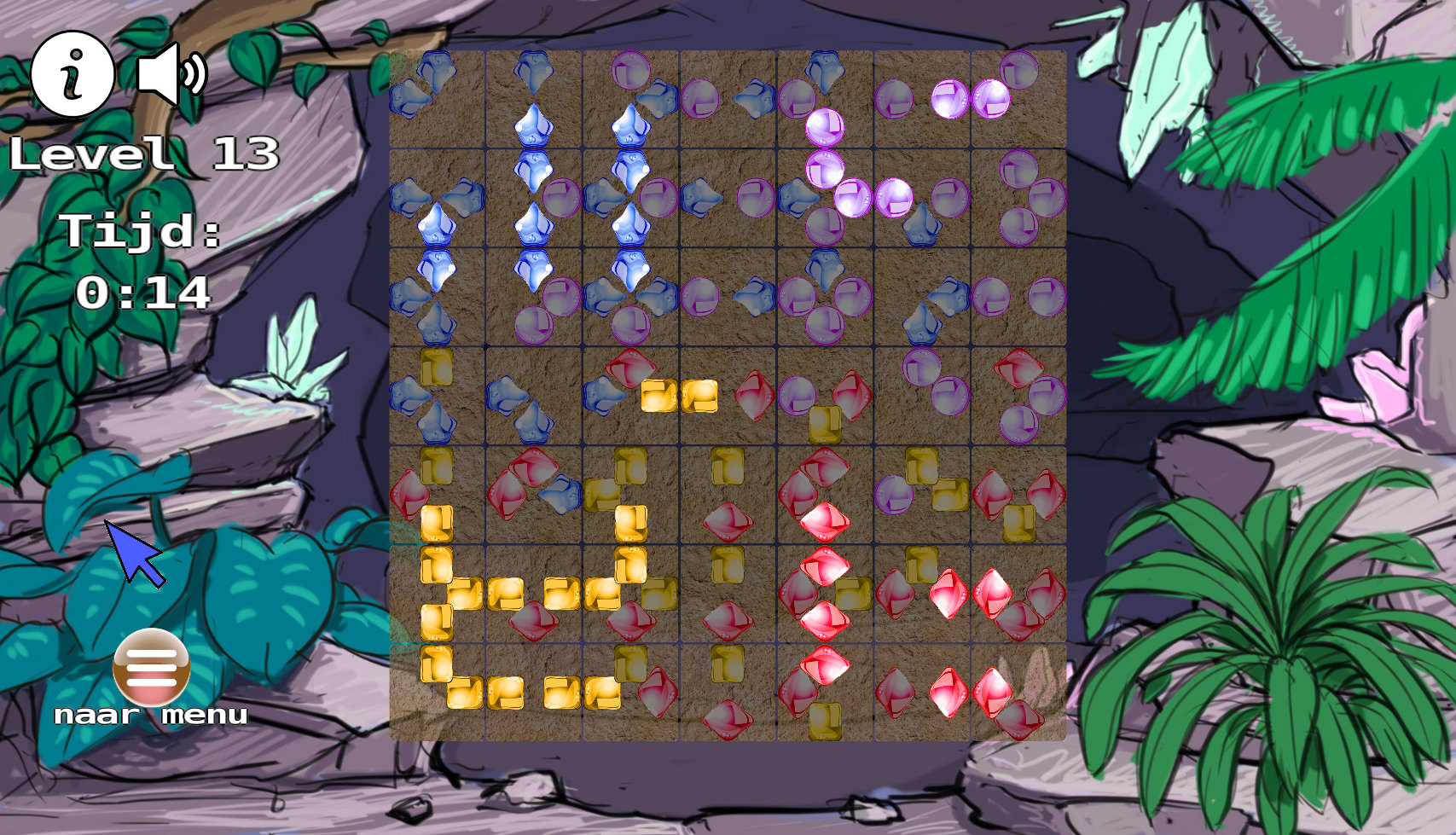

Supplement: Multimedia Appendix 4 [file formative_v8i1e13723_app4.zip › MMA2 - Prototype 3.4.jpg]
